# Medico-Legal Congress

**Published:** 1895-08

**Authors:** 


### Society Notes.

### Medico-Legal Congress—Summer Vacation of 1895—Preliminary Announcement.

The Medico-Legal Society announces a Medico-Legal Congress at or near the City of New York, on the 4th, 5th and 6th of September, 1895 (place to be hereafter announced), open to all students of medical jurisprudence, under the charge of a committee of arrangements, composed as follows: Ex-Surrogate Rastus S. Ransom, Chairman; Clark Bell, Esq., Secretary, 57 Broadway, N. Y.; George Chaffee, M. D., Treasurer, 228 47th St., Brooklyn, N. Y.; Moritz Ellinger, Esq.; Constine J. MacGuire, M. D.; H. W. Mitchell, M. D.; Prof. A. M. Phelps, M. D.

An enrolling fee of $3.00 will be required of each member to aid in the expenses and the publication of the Bulletin of the Congress, to be sent each member free.

A general invitation to all persons interested in the science of medical jurisprudence is extended, and members of the society, active, corresponding or honorary, who wish to enroll will forward their names and the enrolling fee to the secretary or treasurer. A considerable number of papers have already been secured, and the interest taken in the movement warrants the expectation of a large and influential meeting of the friends and students of forensic medicine on the occasion.

The following titles of papers are already received and announced:

1. PSYCHOLOGY AND PSYCHOLOGICAL MEDICINE.

(*a*) INSANITY AND MENTAL MEDICINE—Forbes Winslow, M. D., London, Chairman.

W. B. Fletcher, M. D., Indianapolis, "What Constitutes Unsoundness of Mind."

Frank P. Norbury, M. D., Jacksonville, Ill., "The Mental Symptoms of Premature Sexual Decay."

James R. Cocke, M. D., Boston, Mass., "Latent Hysteria as a Cause of Temporary Mental Disease."

Prof. C. H. Hughes, M. D., St. Louis, Mo., "Paranoia."

Wm. F. Drewry, M. D., Petersburgh, Va., "Simulation of Insanity. A Medico-Legal Study."

G. E. Shuttleworth, M. D., Richmond, England, "Legal Responsibility in Idiotic and Feeble-Minded Persons."

(*b*) INEBRIETY—T. D. Crothers, M. D., Chairman.

Norman Kerr, M. D., London, "What shall we do with the Alcoholic Inebriate, Apparently Insane?"

Lewis Mason, M. D., Brooklyn, N. Y., "Questions of Responsibility in Alcoholic Coma, found on the Street."

Isaac N. Quimby, M. D., Jersey City, N. J., "Alcoholic Anæsthesia a Factor in Crime."

E. C. Mann, M. D., N. Y., "Inebriety and the Opium Habit in the their Relation to Testamentary Capacity."

T. D. Crothers, M. D., "Legal Responsibility in Inebriety."

(*c*) SOCIOLOGY AND CRIMINOLOGY—Hon. Moritz Ellinger, Chairman.

Forbes Winslow, M. D., London, "Suicide Considered as a Mental Epidemic."

Gustave Boehm, Esq., New York City, "Suicide and the Right to Commit It." "Prostitution—The Evil; The Cure; Legislation, etc."

Dan'l R. Brower, M. D., 597 Jackson Boulevard, Chicago, Ill., "Criminality a Disease, its Etiology and Treatment."

Dr. Havelock Ellis, M. D., London, "Sexual Inversion, with Analysis of 36 New Cases."

Prof. Elliott Coues, Washington, D. C., "The Megalomania of H. P. Blavalsky, a Study of Criminal Alienism."

